# Hexicon 2: Automated Processing of Hydrogen-Deuterium Exchange Mass Spectrometry Data with Improved Deuteration Distribution Estimation

**DOI:** 10.1007/s13361-014-0850-y

**Published:** 2014-03-28

**Authors:** Robert Lindner, Xinghua Lou, Jochen Reinstein, Robert L Shoeman, Fred A Hamprecht, Andreas Winkler

**Affiliations:** 1 Department of Biomolecular Mechanisms, Max Planck Institute for Medical Research, Heidelberg, Germany; 2 Heidelberg Collaboratory for Image Processing (HCI), University of Heidelberg, Heidelberg, Germany

**Keywords:** Hydrogen-deuterium exchange, Data analysis, Software, Chromatogram alignment

## Abstract

Hydrogen–deuterium exchange (HDX) experiments analyzed by mass spectrometry (MS) provide information about the dynamics and the solvent accessibility of protein backbone amide hydrogen atoms. Continuous improvement of MS instrumentation has contributed to the increasing popularity of this method; however, comprehensive automated data analysis is only beginning to mature. We present Hexicon 2, an automated pipeline for data analysis and visualization based on the previously published program Hexicon (Lou et al. 2010). Hexicon 2 employs the sensitive NITPICK peak detection algorithm of its predecessor in a divide-and-conquer strategy and adds new features, such as chromatogram alignment and improved peptide sequence assignment. The unique feature of deuteration distribution estimation was retained in Hexicon 2 and improved using an iterative deconvolution algorithm that is robust even to noisy data. In addition, Hexicon 2 provides a data browser that facilitates quality control and provides convenient access to common data visualization tasks. Analysis of a benchmark dataset demonstrates superior performance of Hexicon 2 compared with its predecessor in terms of deuteration centroid recovery and deuteration distribution estimation. Hexicon 2 greatly reduces data analysis time compared with manual analysis, whereas the increased number of peptides provides redundant coverage of the entire protein sequence. Hexicon 2 is a standalone application available free of charge under http://hx2.mpimf-heidelberg.mpg.de.

**Electronic supplementary material:**

The online version of this article (doi:10.1007/s13361-014-0850-y) contains supplementary material, which is available to authorized users.

**Figure:**
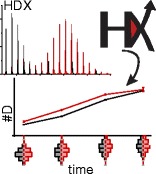
ᅟ

## Introduction

Knowledge of a protein’s structure and dynamics is essential for understanding molecular details of its function. Structural insights at atomic resolution can be obtained from X-ray crystallography, yet protein crystals may be difficult to obtain and provide only limited information about protein dynamics. Nuclear magnetic resonance (NMR) yields both structure and dynamics of a protein in solution; however, size constraints of traditional methods limit the analysis of larger protein complexes or samples that cannot be maintained at high concentrations. In contrast, hydrogen–deuterium exchange mass spectrometry (HDX-MS) requires neither high protein concentrations nor is it limited by protein size [1]. Since the rate of amide hydrogen exchange in the protein backbone depends on solvent accessibility and hydrogen bonding, quantification of the exchange reaction can be used to analyze protein dynamics and secondary structure stability in solution. Although exchange rates alone are difficult to interpret in terms of structural aspects, they are a powerful measure of structural dynamics in the presence of high-resolution structural data or models [1–3]. HDX-MS is particularly suited to compare different functional states of a protein (e.g., to assess the effect of ligand binding on structural dynamics of the protein).

The most established readout of the exchange reaction is bottom-up liquid chromatography (LC) coupled to mass spectrometry (MS) where spatial resolution is obtained from protease digestion prior to chromatographic separation and mass analysis [4]. Such experiments generate multiple two-dimensional LC-MS maps corresponding to the system of interest under different experimental conditions, sampled at a series of D_2_O incubation times. The task is to extract the mass differences of multiple deuterated peptides in all maps relative to those from the undeuterated sample. Manual evaluation, involving peptide sequence assignment, retention time alignment, and centroid mass extraction, is cumbersome, error-prone, and has traditionally been the bottleneck of HDX analysis [1].

Many aspects of HDX-MS data analysis benefit from the mature and more standardized field of proteomics as both types of experiments rely on identification of peptides and correct mapping to the protein of origin. However, since the variable of interest in HDX-datasets is mass difference rather than protein quantity, established proteomics pipelines are not applicable to HDX-analysis; thus, manual intervention or specialized algorithms are required.

Several solutions exist to facilitate individual parts of the workflow, most of which are tools for the calculation of deuteration distributions [5–8] or centroids [9, 10]. A specialized chromatogram alignment tool was shown to improve feature matching across different LC-MS maps [11]. Implementations are not always readily available [5, 11], not fully documented, command-line based [7, 8], or available as spreadsheet macro [9, 10]. Since these tools require different input and produce nonstandard output, combining them into a fully automated analysis pipeline is difficult.

Software covering the entire range of data analysis tasks has only recently become publicly available. Hydra [12] was one of the first standalone applications capable of providing a complete workflow and deuteration distribution estimation; however, it lacked a robust default pipeline. Currently, this package is further developed under the name Mass Spec Studio [13]. ExMS [14] is a suite of MATLAB scripts that extract and validate peaks predicted from a list of MS/MS-validated search peptides. Commercial dependencies and the lack of a full graphical user interface (GUI) are the major drawbacks of this algorithmically sound workflow. HDXFinder [15] is a recently published web-based analysis server, however increasing data volumes and local computing power clearly favor standalone solutions. HDX Workbench [16] is a standalone application that has evolved from the web-based pipeline HD Desktop [17] and combines a complete analysis workflow with a GUI offering rich data visualization and manipulation capabilities. The workflow is geared towards high-resolution mass spectra but currently only supports native data from Thermo Scientific instruments. Hexicon [18], another standalone solution, was the first to apply de novo feature detection rather than relying on a predefined peptide list, in the attempt to increase protein sequence coverage. A regularized regression model (non-greedy, iterative template-based peak picker, NITPICK [19]) allows identification of peptides in the undeuterated reference and is used to estimate the distribution of deuteration states in each peptide at each D_2_O incubation time point. The ability to estimate deuterium incorporation distributions provides additional insight into the exchange mechanism and allows detection of otherwise underestimated differences in exchange behavior under different conditions [20, 21].

Despite its strong algorithmic foundations, Hexicon has some shortcomings that have precluded its widespread use. The runtime of NITPICK feature detection is effectively quadratic with respect to the spectrum size and, therefore, requires segmentation of the LC-MS map both in the LC and the MS dimensions for manageable runtime. For this purpose, Hexicon applies watershed segmentation, which resamples the map into evenly spaced bins and is extremely sensitive to variation of the intensity baseline, limiting the range of amenable experimental settings. It was further shown that the L1-regularized regression applied by NITPICK yields overly sparse estimates of the deuteration distribution in some cases [21]. Although Hexicon reads spectra in the open mzXML format, it cannot be used for many HDX experiments, as its quality control classifier was trained on data from a specific QSTAR Pulsar (Applied Biosystems, Darmstadt, Germany) instrument, and performs poorly on data collected on other instruments.

We tackle several limitations of Hexicon by embedding the NITPICK algorithm into a workflow that includes chromatographic alignment and targeted feature extraction from maps containing deuterated peptides rather than repeated de novo feature detection in deuterated samples and greedy instrument-specific correspondence matching. In addition, deuteration distribution estimation now employs a smoothing rather than a sparseness-promoting algorithm. Hexicon 2 introduces novel features to address problems of current HDX experimentation, including fast handling of high-resolution LC-MS data, internal mass calibration, processing of multi-protein mixtures, and convenient automated and user-supervised resolution of multiple sequence assignments. Downstream processing of HDX data is facilitated by a result browser, which supports manual and automated data filtering as well as a number of frequently used visualization options.

In this work, we present Hexicon 2, a fully automated workflow for accurate analysis of HDX/LC-MS data, to exploit the full potential of high-resolution mass spectrometry data [22] and to accommodate a larger number of experimental scenarios. Hexicon 2 outperforms its predecessor in terms of speed and accuracy of deuteration distribution estimation and significantly increases the peptide coverage compared with manual HDX analysis. The Hexicon 2 program provides a robust and instrument-independent solution for processing and presentation of HDX-MS data in a user-friendly graphical environment.

## Experimental

High-resolution HDX data of the AppA–PpsR system from *Rhodobacter sphaeroides* [23, 24] was used to benchmark Hexicon 2. Recombinant protein expression, purification and complex formation as well as continuous labeling HDX have been described previously [24]. Briefly, AppA (200 μM) and PpsR (400 μM) as well as the preformed AppA–PpsR_2_ complex (200 μM) were diluted 20-fold in D_2_O buffer containing 10 mM CHES pD 9.5, 150 mM NaCl, and 5% glycer(ol-d3). Aliquots (20 pmol AppA or AppA–PpsR_2_, 40 pmol PpsR) were removed after 15, 60, 300, and 1200 s and the reaction was quenched in ice-cold aqueous 200 mM ammonium formate buffer pH 2.6, directly followed by injection into the LC-MS setup (Shimadzu Prominence HPLC, Shimadzu, Duisburg, Germany). The chromatography setup was cooled to 0.5°C in a water/ethylene glycol bath and consisted of a 2 cm guard column (Discovery Bio C18, Supelco, Bellefonte, PA) for desalting and a 10 cm analytical column (Discovery Bio Wide Pore C18 10 cm × 1 mm; 3 μm particle size, Supelco, Bellefonte, PA). The pepsin column (Applied Biosystems, Darmstadt, Germany) was operated at 10°C for on-line proteolysis during a contact time of 1 min. Separation of peptides was achieved by a 20 min acetonitrile gradient (15%-50% in H_2_O with 0.6% formic acid) prior to injection into a maXis UHR-TOF (Bruker, Bremen, Germany) mass spectrometer. LC-MS spectra were acquired at a scan rate of 0.5 Hz and filtered with an intensity threshold of 100 counts. Peak detection was performed with the Apex algorithm provided by the acquisition software (Compass DataAnalysis 4.0, Bruker, Bremen, Germany), using a peak width setting of 0.02 Da.

## Methods

### Algorithmic Strategy of Hexicon 2

**Figure 1:**
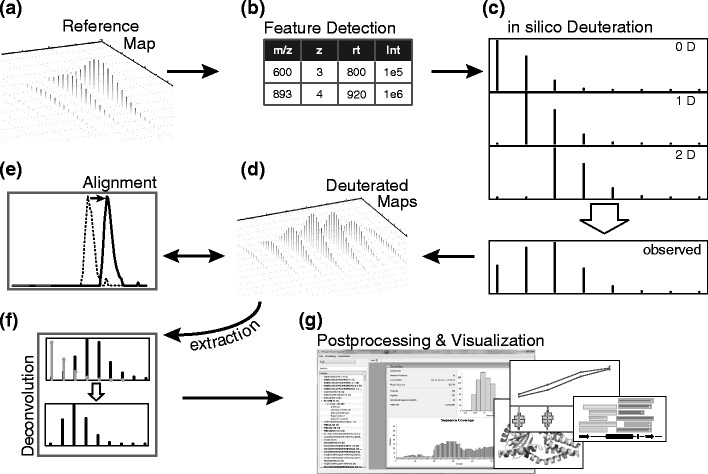
Hexicon 2 workflow. Starting from the undeuterated reference LC-MS map (**a**) features are extracted using the NITPICK algorithm and candidate peptide sequences are assigned (**b**). Detected features are deuterated in silico (**c**) and matching signals are searched for in LC-MS maps containing deuterated peptides (**d**). An alignment (**e**) is generated to predict retention times at which deuterated peptides elute in order to narrow down the search space from which corresponding peaks are extracted. Iterative deconvolution (**f**) is applied to estimate the distribution of deuteration states of each extracted peptide. The result browser (**g**) provides several options for result post-processing, visualization, and export as vector graphics or raw data

Hexicon [18] was restructured in Hexicon 2 to optimize runtime and accuracy for the analysis of high-resolution hydrogen-deuterium exchange mass spectrometry data. Figure 1 shows the new workflow of Hexicon 2. Details of the individual analysis steps are given below; in summary, the NITPICK algorithm [19] is used to detect peptide signals in the mass spectra (Fig. 1a, b), followed by sequence assignment and in silico deuteration (Fig. 1c). LC-MS maps of deuterated peptides (Fig. 1d) are then scanned for predicted deuterated masses and a retention time mapping is inferred from this information. This alignment defines a time window during which each deuterated peptide is expected to elute (Fig. 1e). Hexicon 2 takes full advantage of high-resolution data by extracting peak intensities within resolution-dependent regions predicted by in silico deuteration and chromatographic alignment, allowing for robust estimation of deuteration distributions using an iterative deconvolution procedure (Fig. 1f). Hexicon 2 provides a result browser that allows convenient post-processing as well as export of publication quality figures and spreadsheets for customized downstream analysis (Fig. 1g).

### Feature Detection

NITPICK has been described in detail previously [19]. Briefly, the algorithm casts the problem of feature detection (i.e., the extraction of monoisotopic masses, corresponding charge states and abundances) as model generation and regression problem in which the observed mass spectrum ***s*** is modeled as a linear combination of isotope patterns *ϕ* (Equation 1) from *K* peptides present at relative concentrations *β*.

1 $$ \boldsymbol{s}={\displaystyle \sum_{i=1}^K{\beta}_i\ast {\boldsymbol{\phi}}_i}=\boldsymbol{\Phi} \boldsymbol{\beta} $$

Since neither the peptides nor their isotope patterns are known in advance, NITPICK generates an over-complete set of candidate isotope patterns using an improved Averagine model [25] of all peaks exceeding a user-specified signal to noise ratio (SNR). Columns of the regression matrix *Φ* that are justified by the spectrum are then selected using a non-negative LASSO [26] model (Equation 2):

2 $$ \widehat{\beta}= \arg \min {\left\Vert \boldsymbol{s}-\boldsymbol{\Phi} \boldsymbol{\beta} \right\Vert}^2+\lambda {\displaystyle \sum_{i=1}^K\left|{\beta}_i\right|}\kern.33em s.t.\kern0.5em {\beta}_i\ge 0 $$

The regularization parameter *λ* controls the trade-off between the sum of squares term and the L1 norm of the coefficient vector β. Higher values of *λ* will trade the goodness of fit for sparseness of the solution, and optimal choice of λ is vital for the quality of the model. NITPICK uses non-negative least angle regression (LARS) [27] and terminates when a minimum Bayesian information criterion [28] is found. LARS is an algorithm to solve LASSO problems without explicitly searching for an optimal *λ*, thereby greatly reducing the runtime [27]. Feature selection runs in one dimension and has a runtime complexity of $\mathcal{O}\left(\left|\boldsymbol{s}\right|{\left|\varPhi \right|}^2\right)$, where |***s***| and |*Φ*| indicate the size of the spectrum and the number of models (columns) in the regression matrix, respectively. Hexicon 2 improves runtime and sensitivity by dividing each scan into segments of peptides with non-overlapping isotopic envelopes, followed by merging of features from adjacent scans into elution profiles. Here, it is assumed that each peptide elutes in a number of subsequent scans containing no more than a user-defined number of internal gaps (i.e., scans in which the feature was not detected).

### Sequence Assignment

Hexicon 2 assigns possible peptide sequences to each eluting peptide based on the attainable mass precision. Confirmed peptide sequences (e.g., from MS/MS sequencing) are the preferred source of sequence information, to which peptides from unspecific in silico digestion of the user-defined protein sequence are added. In high-resolution settings, the loss of accuracy through poor calibration may be significantly larger than the resolution (Supplemental Figure 1); hence, Hexicon 2 uses dominant Δ*m*/*z* values encountered during sequence assignment to internally recalibrate the spectra. If MS/MS confirmation of a peptide sequence is absent, all sequence assignments within the instrument precision range are kept and reported. Many ambiguous sequence assignments can be resolved by taking into account protease specificity: by default, Hexicon 2 assigns pepsin cleavage scores based on the study carried out by Hamuro and colleagues [29] but other score sets for different proteases can be defined by the user. Protease scores for each peptide are reported in the analysis output and help to judge the quality of sequence assignments in the absence of MS/MS identification.

### Chromatogram Alignment

The newly implemented alignment algorithm differs from general-purpose LC-MS alignment: it predicts which features to expect at a given retention time rather than matching previously extracted features. Thus, Hexicon 2 utilizes chromatogram alignment not only to match deuterated species to the reference but also to reduce the number of searched spectra for improved runtime and specificity.

Initially, an alignment candidate retention time mapping is created by probing all masses expected after deuteration within boxes adjusted to the instrument’s resolving power, using the fast box intersection library libfbi [30]. For each peptide, retention times containing a required number of consecutive isotope peaks are mapped to the retention time detected in the undeuterated reference LC-MS map. The generated candidate map contains randomly scattered false alignments and a continuous region of increased density representing the correct alignment. A coarse linear estimate for this region is made and candidate alignments with a regression residual larger than one standard deviation are removed. Piecewise linear regression is then applied to generate a smooth nonlinear mapping of reference retention times to those from the current map. To account for non-zero elution peak width, mapped retention time centroids are extended by the reference peak width. Mapping uncertainty is taken into account by a tolerance window, which is locally adjusted based on residuals from the piecewise linear regression model such that the alignment of regions with higher mapping uncertainty is more permissive. Online Resource 1 contains a comprehensive description of the alignment procedure.

### Deuteration Distribution Estimation

High mass resolution alleviates the problem of unresolvable overlapping peaks, which is why many current algorithms rely on direct intensity extraction rather than computationally more expensive model unmixing [14, 16, 22]. Hexicon 2 follows a similar strategy and extracts peak intensities in predicted *m*/*z* regions. If a required number of consecutive isotopes with appreciable intensity are found, the isotope pattern is deconvoluted using the natural isotope pattern as point spread function, yielding the deuterium incorporation distribution. Unregularized approaches to deconvolution (e.g., using the convolution theorem [7] or unconstrained least squares [8]) lead to solutions that are very sensitive to noise [5, 31]. Entropy-maximizing regularization imposes a bias towards uniformly distributed deuteration and has been shown to provide smooth and condensed distributions if properly regularized [5]. However, nonlinear optimization and the search for regularization make maximum entropy methods computationally inefficient. Gold’s iterative deconvolution algorithm assigns positive distribution coefficients using a gradient descent and favors smooth solutions similar to maximum entropy regularization [32]. The distribution estimation algorithm employed in Hexicon 2 is based on the implementation of Gold deconvolution from the ROOT toolkit developed at the CERN [33, 34]. The implementation takes advantage of matrix shapes and substantially reduces the number of computations as well as memory requirements by using pre-computed constants [33].

Typical HDX analysis determines the mass centroid shift between the deuterated and the native isotope pattern of a peptide. This value corresponds to the centroid of the deuterium incorporation distribution determined by Hexicon 2. If data for a 100% deuterated control (e.g., unfolded protein) is provided, centroid values can be corrected for back-exchange as described by Zhang and Smith [4]. Uncorrected relative deuteration differences can be used for comparative experiments carried out under identical LC-MS conditions [9].

## Results and Discussion

### Runtime and Memory Usage

High resolution mass spectrometers generate data at high density (e.g., a median of 290,000 data points per scan with a Bruker maXis UHR-TOF) and sophisticated methods for peak detection are frequently part of the instrument’s data acquisition and analysis software (e.g., Compass DataAnalysis 4.0, Bruker). Such processing eliminates instrument-specific peak shapes and reduces the data volume by a factor of 10–20. Hexicon 2 is able to detect peptides in line spectra and together with its divide-and-conquer progression scheme, even large quantities of data are processed with a memory footprint that can be provided by a desktop PC. Runtime and memory usage (Table 1) were recorded on a SuSE 11.4 (Linux 2.6.37 x86_64) workstation with a 2.8 GHz CPU and 24 GB RAM for an HDX experiment investigating protein interactions in the AppA–PpsR regulatory system from *Rhodobacter sphaeroides* [24]. Note that the mzXML file sizes and numbers of data points refer to the reference map in which feature extraction was carried out, however, total runtime and peak memory usage are shown for the analysis of a complete experiment which contained 12 additional maps of similar size from four D_2_O incubation time points measured in triplicate.

**Table 1:** Runtime and Memory Footprint for Hexicon 2 Analysis of Different Datasets. Note that File Size and Number of Data Points Refer to the Reference Dataset Only. For Each Experiment, 12 Additional Maps of Similar Size Were Analyzed Using the Given Resources

Dataset	File size	Data points	Feature candidates	Runtime	Memory
AppA	98 MB	9.50E + 06	1734	5:00 min	550 MB
PpsR	93 MB	9.00E + 06	1868	5:50 min	470 MB
AppA–PpsR	106 MB	1.00E + 07	3106	8:00 min	570 MB

### Protein Coverage and Sequence Assignment Quality

Hexicon 2 increases protein sequence coverage and redundancy by running de novo feature detection in the reference LC-MS map and by assigning candidate sequences also to peptides without MS/MS confirmation. Manual analysis of AppA and PpsR HDX data was based on peptide lists generated from 1 h LC-MS/MS runs with automated precursor selection and yielded mostly non-redundant coverage of 91% and 97% of the AppA and the PpsR protein sequence with 22 and 39 peptides, respectively. Hexicon 2 analysis yielded full deuteration time series for 253 peptides of AppA (100% coverage) and 359 peptides of PpsR (100% coverage) after automated post-processing and manual removal of poor quality results. Table 2 shows coverage statistics for all analyzed datasets as well as the number of intersecting peptides for comparative analysis. In total, analysis of the experiment required seven runs of Hexicon 2: two for AppA (dark and light), two for AppA peptides in the AppA–PpsR_2_ complex (dark and light), one for free PpsR, and two for PpsR peptides in the AppA–PpsR_2_ complex (dark and light). After suitable processing parameters had been determined based on the chromatographic profile and the desired sensitivity, analysis of seven datasets, including manual curation and across-dataset comparisons, was accomplished in less than one working day. Hexicon 2 not only increased sequence coverage but also the redundancy of 15 features per amide on average for all analyzed experiments allowed additional quality control and resolution enhancement from overlapping peptides (Supplemental Figures 2 and 3).

**Table 2:** Coverage Statistics for Hexicon 2 Analysis of the Interaction of AppA (44 kDa) with PpsR (51 kDa) and the Influence of Light. HDX Data of Seven Conditions (Four for AppA and Three for PpsR) Were Analyzed. “C” Indicates that the Respective Protein Was Analyzed after Labeling of the AppA–PpsR_2_ Complex. Statistics of Comparative Analyses Indicate Coverage Obtained from the Intersection of Datasets (e.g., Features Retrieved in Data from Both the Free and Complex-Bound Protein, to Study Complex Formation). Coverage Denotes the Proportion of Amides Covered by at Least One Feature; Redundancy Denotes the Median Number of Features Covering One Amide. All Statistics Were Obtained after Automated Filtering and Manual Removal of Poor Quality Results

Dataset	Features	Peptides	Coverage	Redundancy
AppA				
Dark	368	253	100.0 %	13×
Light	431	291	100.0 %	17×
C-dark	288	200	98.8 %	12×
C-light	267	186	98.8 %	12×
PpsR				
Dark	546	359	100.0 %	16×
C-dark	475	322	100.0 %	20×
C-light	443	315	100.0 %	17×
Comparative: complex formation				
AppA	192	144	98.3 %	7×
PpsR	420	261	99.1 %	11×
Comparative: illumination				
AppA alone	352	236	98.3 %	11×
C (AppA)	261	181	98.8 %	11×
C (PpsR)	439	290	99.6 %	13×
Comparative: across all datasets				
AppA (4)	205	140	95.8 %	7×
PpsR (3)	403	248	98.7 %	11×

**Figure 2:**
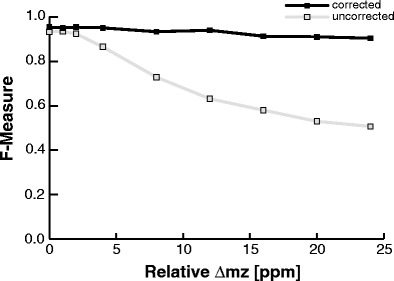
Sequence assignment quality with and without internal recalibration. Four hundred ninety-seven features of PpsR were used as gold standard to simulate LC-MS spectra. Different calibration errors from 1 to 24 ppm were introduced artificially and Hexicon 2 was run for feature detection and sequence assignment with (black) and without (gray) internal recalibration of *m/z* values. The F-measure is the harmonic mean of sequence assignment precision and recall

Finite instrument precision, calibration uncertainty, and the large number of isobaric peptides that are generated by in silico digestion of a protein are points of concern for mass-based sequence assignment. Therefore, Hexicon 2 performs internal mass calibration, determines mass precision, and estimates the false discovery rate (FDR) to minimize the number of false sequence assignments and to inform the user about assignment uncertainty. We assessed the benefits of internal calibration by simulating theoretical spectra for 373 peptides (497 features) and artificially introducing a calibration error. Both sequence assignment precision and sensitivity declined considerably when calibration errors exceeded the instrument precision of 4 ppm unless correction was applied (Fig. 2). Considering the AppA–PpsR_2_ complex dataset as most extreme example at hand, we found a relative calibration error of 16 ppm, which in our simulation corresponded to a false sequence assignment rate of nearly 50% in the absence of recalibration as opposed to less than 8% when internal calibration was carried out.

It has been shown that such a statistical approach combined with protease specificity scoring can resolve many sequence assignment conflicts in the absence of MS/MS identification [35]. Nevertheless, sequence assignment relying only on mass shows false discovery rates ranging from 10% to 15% in realistic experimental scenarios; hence, MS/MS confirmation is required for absolute confidence. In such cases, MS1 sequence assignments generated by Hexicon 2 can assist in generating precursor lists for MS/MS confirmation of critical peptides.

### Retention Time Alignment

**Figure 3:**
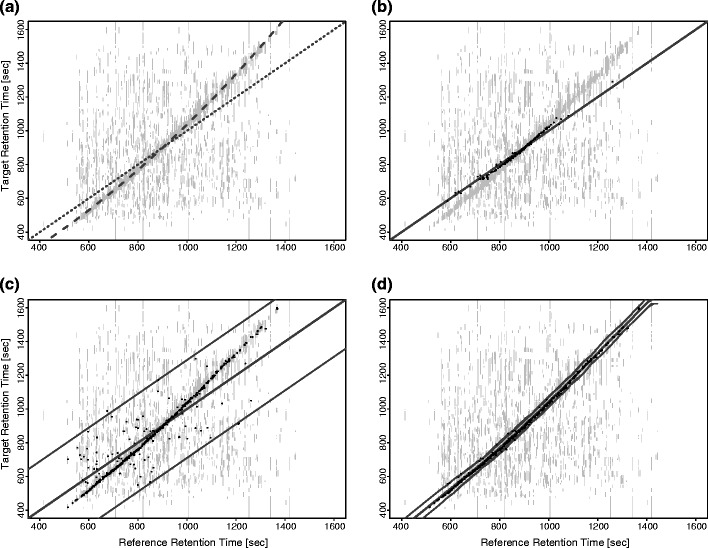
Retention time alignment. A nonlinear retention time shift [(**a**), dashed line] was introduced into an almost perfectly aligned [(**a**), dotted line] map. This resulted in retention time differences between –80 and +290 s. Gray lines in the background indicate retention times at which undeuterated reference masses match simulated deuterated masses. Dots indicate retention time coordinates at which features were detected in both the undeuterated and the deuterated map (i.e., an aligned pair of features). Feature pairs off the indicated shift can be considered false positives. (**b**) Recovered features in the absence of alignment (gray line indicates 1:1 mapping), using a 30 s search window. (**c**) Recovered features in the absence of alignment using a 290 s search window to accommodate all shifted features. Gray lines indicate the 1:1 mapping and the boundaries of the search window. (**d**) Recovered features using the alignment algorithm implemented in Hexicon 2. Gray lines indicate the retention time mapping and the locally adjusted search window

Hexicon 2 implements a nonlinear retention time alignment that assigns an expected elution time range to each reference peptide. For the purpose of demonstration, we introduced a nonlinear retention time shift into a previously almost perfectly aligned LC-MS map by multiplying original scan times with ascending values between 0.8 and 1.2 (Fig. 3). This resulted in distortion of elution peaks and a retention time difference ranging from –80 to 290 s with respect to the reference. The intersection algorithm created a visible region of increased density that corresponded well with the simulated offset (Fig. 3a). Following banding and piecewise linear regression, the alignment matched the simulated retention times up to an accuracy of 3.3 ± 8.5 s and added an empirical tolerance window of ±30 s on average (Fig. 3d) as opposed to the 290 s window that would be necessary to accommodate all shifted features in the absence of alignment (Fig. 3c).

Hexicon 2 analysis using alignment was superior to a fixed retention time window both in terms of sensitivity and specificity. When window size was chosen too small (Fig. 3b), a large number of features remained unmatched, whereas a window size large enough to accommodate all shifted features (Fig. 3c) resulted in a visibly increased false positive rate compared with the analysis using alignment (Fig. 3d). In this respect, it should be emphasized that despite the software’s ability to process LC-MS maps with significantly different elution profiles, artefacts from inconsistent back-exchange between deuterated samples with different retention times are not corrected for.

### Deuteration Centroid and Distribution Estimation

Deuteration centroids extracted by Hexicon 2 were compared with semi-automated analysis using the spreadsheet macro HX-Express [9], which served as a gold standard. In total, 96 peptides from PpsR (33 free PpsR, 31 AppA-bound dark state, 32 AppA-bound light state) and 74 peptides from AppA (18 of free AppA in the dark and 19 in the light, and 19 and 18 of PpsR-bound AppA in dark and light state, respectively) were commonly found in manual and automated analyses. The median deviation of automated analysis from manual extraction was 0.12 Da (3.1% of total deuteration), with 90% of automated analysis yielding a value within 10% of manual analysis and 95% differing by less than 22% (Supplemental Figure 4; underlying data in Supplemental Figures 5–8). In most cases, failure of automated extraction using Hexicon 2 occurred because of isotope patterns with multiple overlapping peaks such that peaks were not distinguishable at the resolution of 40,000 FWHM.

Although further improvements in mass resolution and chromatographic separation will make such problems less frequent, data analysis solutions using regression or other mixture models outperform the commonly used extraction-based approaches in the case of highly overlapping distributions. The large number of peptides and possible deuteration states, however, render mixture modeling strategies impractical unless a precise prediction of which signals to expect within the spectrum can be made.

**Figure 4:**
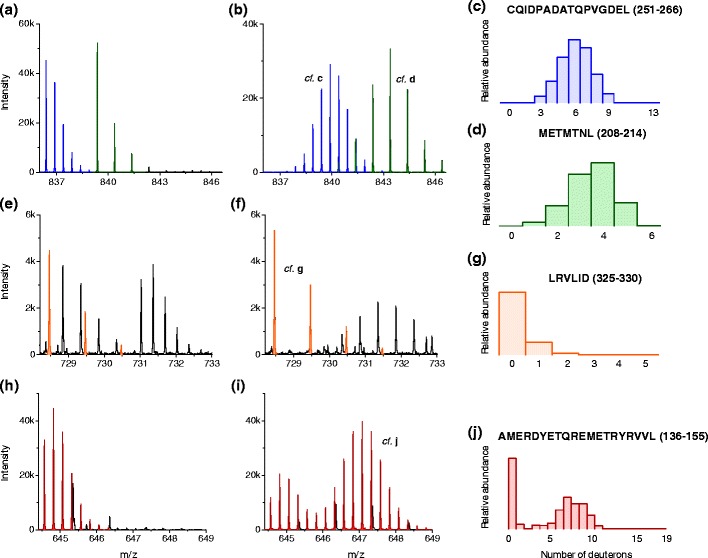
Deuteration distribution estimation. Mass spectra of undeuterated [left column, panels (**a**), (**e**), (**h**)] and corresponding deuterated (middle column) peptides of PpsR [panels (**b**), (**f**)], and the AppA–PpsR_2_ complex [panel (**i**)] after 60 s D_2_O incubation are shown together with corresponding estimates of the deuterium incorporation distribution [right column, panels (**c**), (**d**), (**g**), (**j**)]. Panels (**a**) and (**b**) show spectra corresponding to the peptides CQIDPADATQPVGDEL (blue) and METMTNL (green), which have overlapping deuterated isotope patterns. Hexicon 2 successfully separates the isotope patterns and creates smooth deuteration distribution estimates for both peptides [panels (**c**) and (**d**)]. The spectra corresponding to the peptide LRVLID exhibit low signal intensity both in the reference and the deuterated sample [panels (**e**) and (**f**), respectively] and overlap with other peaks. The bimodal deuterated spectrum corresponding to the peptide AMERDYETQREMETRYRVVL [panels (**h**) and (**i**)] is correctly detected as one isotope distribution, and deconvolution (**j**) reveals that the spectrum is composed of one undeuterated population and one that is centered around nine incorporated deuterons

Hexicon included a method for deuteration distribution estimation (i.e., deconvolution of the natural and deuteration-induced isotope patterns). Lou and colleagues showed that the NITPICK algorithm is superior to alternative deconvolution methods [18]; however, it was also observed that L1-regularization leads to overly sparse estimates and misses bimodal distributions in some cases [21]. The newly introduced boosted iterative deconvolution algorithm in Hexicon 2 drives the result towards a smooth estimate similar to maximum entropy regularization, which was shown to be well-suited for the distribution estimation problem [5, 32]. Figure 4 shows representative reference and deuterated mass spectra containing overlapping isotope patterns from multiple peptides, low intensity peptides, or such exhibiting bimodal exchange behavior together with the corresponding deuteration distribution estimates. In order to quantify the advantage of iterative deconvolution used in Hexicon 2 over NITPICK, we inspected deuteration distributions of 242 PpsR peptides that were assigned similar deuteration centroid values (no more than 10% deviation) by both algorithms. Distributions were blindly classified as realistic or unrealistic based on visual inspection of the underlying mass spectra. Gold deconvolution yielded 230 realistic and 12 unrealistic estimates, whereas NITPICK performed worse at 192 and 50 realistic and unrealistic estimates, respectively. Nineteen out of the 50 unrealistic distributions returned by NITPICK assigned the entire spectrum to only one deuteration state and in 13 cases contained an overly condensed distribution that overestimated deuteration states near the centroid (e.g., Supplemental Figure 9). NITPICK generally produced narrower distribution estimates, which bears the risk of not identifying correlated exchange regimes [36].

### Post-Processing and Visualization

**Figure 5:**
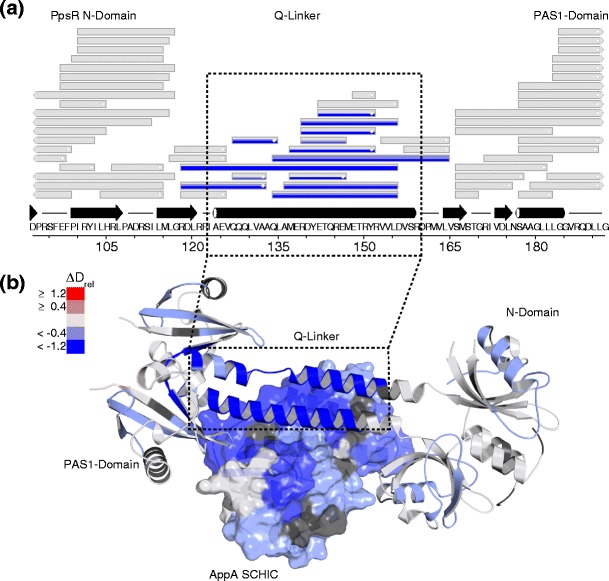
Examples of protein level plots generated using Hexicon 2 results. Both panels show deuteration differences between free AppA and PpsR and both proteins in the AppA–PpsR_2_ complex. (**a**) Part of a PpsR peptide map generated by the Hexicon 2 result browser. Each box represents one peptide and can contain multiple colors indicating the difference in relative deuteration (∆D_rel_) at each incubation time, bottom-up. Red color indicates higher relative deuteration in the AppA–PpsR_2_ complex compared with free PpsR and blue color indicates lower relative deuteration in the complex. A secondary structure prediction can be loaded from a CSV file and displayed above the sequence. (**b**) Mapping of the difference in relative deuteration after 15 s of D_2_O incubation on a three-dimensional crystal structure model of the AppA–PpsR_2_ core complex [26] using PyMol [37]. AppA is shown in a surface representation, PpsR as a cartoon. The marked helical linker region of PpsR shows significant protection from exchange upon complex formation as it is part of the complex interface, which is buried and stabilized by binding to AppA

The graphical user interface (GUI) of Hexicon was overhauled to enhance user experience and to accommodate new features, such as the handling of experimental replicates, the use of a fully deuterated control, or the addition of custom protein modifications. Besides the core workflow, Hexicon 2 comes with a result browser that features common data visualization and filtering tasks. Peptides are grouped by sequence in a searchable list from which the user can plot deuteration time series for one or multiple peptides, including deuteration distributions and interactive measurement of deuteration differences (Supplemental Figure 10). On the protein scale, peptide maps and secondary structures can be plotted and color-coded with deuteration values or differences in deuteration levels of two experiments. Mapping on three-dimensional structural models is realized by the creation of a whitespace-separated table containing residue number, name, and deuteration value, which can be fed into the Python script data2bfactor for PyMol [37], a commonly used program in the field of protein structure visualization. Figure 5 shows a part of a peptide map of PpsR and a crystal structure model of the AppA–PpsR_2_ core complex [24], both color-coded to indicate the deuteration differences between the AppA–PpsR_2_ complex in comparison with free AppA and PpsR. One additional example for the use of Hexicon 2 for data analysis and generation of publication-quality figures can be seen in reference [38] in which HDX data were entirely processed using Hexicon 2.

We anticipate that downstream processing and visualization are parts of the workflow that require the largest degree of customization. Therefore, Hexicon 2 retains the option to export relevant data into a comma-separated file for ease of processing with spreadsheet software or statistical tools.

## Conclusions

Hydrogen–deuterium exchange mass spectrometry is a powerful method to study protein structural dynamics in solution. Data analysis has traditionally been the bottleneck for the application of HDX, and with Hexicon 2 we contribute a flexible and fast solution to the rapidly growing field of HDX analysis software. Hexicon 2 retains and improves unique features from its predecessor Hexicon [18], including de novo feature detection, sequence assignment, and deuteration distribution estimation. Further features, such as retention time alignment, protein modifications, and optional back-exchange correction, extend the range of applications for Hexicon 2. The ability to process line spectra and to read the widely used mzXML format allow data from various instruments to be processed after peak picking. The result browser facilitates post-processing and filtering in a semi-automated fashion such that instrument-specific quality control is no longer required. Deuteration time series and distributions can also be exported as comma-separated tables for customized processing or visualization using spreadsheet software or table-based analysis tools.

The modular architecture of the Hexicon 2 workflow allows individual parts to be adapted or replaced in a situation-specific manner. Since retention time prediction now allows precise model generation, we are experimenting with smoothing mixture models for deuteration distribution estimation in future releases. Such models are expected to perform better for highly complex spectra at manageable computational burden and will further push the size and complexity limits of HDX experiments.

Hexicon 2 is available free of charge upon request as a standalone application for Microsoft Windows. In conclusion, our workflow greatly facilitates and accelerates the analysis of HDX-MS experiments and may promote the development of new applications of the H-D exchange reaction, which have been previously deemed too laborious.

## Electronic supplementary material

Below is the link to the electronic supplementary material.

ESM 1 (PDF 935 kb)

## References

[1] Engen JR (2009). Analysis of protein conformation and dynamics by hydrogen/deuterium exchange MS. Anal. Chem..

[2] Krishna MMG, Hoang L, Lin Y, Englander SW (2004). Hydrogen exchange methods to study protein folding. Methods.

[3] Marcsisin SR, Engen JR (2010). Hydrogen exchange mass spectrometry: what is it and what can it tell us?. Anal. Bioanal. Chem..

[4] Zhang Z, Smith DL (1993). Determination of amide hydrogen exchange by mass spectrometry: a new tool for protein structure elucidation. Protein Sci..

[5] Zhang Z, Guan S, Marshall AG (1997). Enhancement of the effective resolution of mass spectra of high-mass biomolecules by maximum entropy-based deconvolution to eliminate the isotopic natural abundance distribution. J. Am. Soc. Mass Spectrom..

[6] Palmblad M, Buijs J, Håkansson P (2001). Automatic analysis of hydrogen/deuterium exchange mass spectra of peptides and proteins using calculations of isotopic distributions. J. Am. Soc. Mass Spectrom..

[7] Hotchko M, Anand GS, Komives EA, Ten Eyck LF (2006). Automated extraction of backbone deuteration levels from amide H/2H mass spectrometry experiments. Protein Sci..

[8] Chik, J.K., van der Graaf, J.L., Schriemer, D.C.: Quantitating the statistical distribution of deuterium incorporation to extend the utility of H/D exchange MS data. Anal. Chem. **78**, 207–214 (2006) 10.1021/ac050988l 16383329

[9] Weis DD, Engen JR, Kass IJ (2006). Semi-automated data processing of hydrogen exchange mass spectra using HX-Express. J. Am. Soc. Mass Spectrom..

[10] Guttman M, Weis DD, Engen JR, Lee KK (2013). Analysis of overlapped and noisy hydrogen/deuterium exchange mass spectra. J. Am. Soc. Mass Spectrom..

[11] Venable JD, Scuba W, Brock A (2013). Feature-based retention time alignment for improved HDX MS analysis. J. Am. Soc. Mass Spectrom..

[12] Slysz GW, Baker CA, Bozsa BM (2009). Hydra – software for tailored processing of H/D exchange data from MS or tandem MS analyses. BMC Bioinforma..

[13] Burns KM, Rey M, Baker CAH, Schriemer DC (2013). Platform dependencies in bottom-up hydrogen/deuterium exchange mass spectrometry. Mol. Cell. Proteom.

[14] Kan, Z.-Y., Mayne, L., Sevugan Chetty, P., Englander, S.W.: ExMS: data analysis for HX-MS experiments. J. Am. Soc. Mass Spectrom. **22**, 1906–1915 (2011) 10.1007/s13361-011-0236-3 PMC3398505 21952778

[15] Miller DE, Prasannan CB, Villar MT (2011). HDXFinder: automated analysis and data reporting of deuterium/hydrogen exchange mass spectrometry. J. Am. Soc. Mass Spectrom..

[16] Pascal BD, Willis S, Lauer JL (2012). HDX workbench: software for the analysis of H/D exchange MS data. J. Am. Soc. Mass Spectrom..

[17] Pascal BD, Chalmers MJ, Busby SA, Griffin PR (2009). HD desktop: an integrated platform for the analysis and visualization of H/D exchange data. J. Am. Soc. Mass Spectrom..

[18] Lou X, Kirchner M, Renard BY (2010). Deuteration distribution estimation with improved sequence coverage for HX/MS experiments. Bioinformatics.

[19] Renard BY, Kirchner M, Steen H (2008). NITPICK: peak identification for mass spectrometry data. BMC Bioinforma..

[20] Zhang J, Ramachandran P, Kumar R, Gross ML (2013). H/D exchange centroid monitoring is insufficient to show differences in the behavior of protein states. J. Am. Soc. Mass Spectrom..

[21] Kreshuk A, Stankiewicz M, Lou X (2011). Automated detection and analysis of bimodal isotope peak distributions in H/D exchange mass spectrometry using HeXicon. Int. J. Mass Spectrom..

[22] Kazazic S, Zhang HM, Schaub TM (2010). Automated data reduction for hydrogen/deuterium exchange experiments, enabled by high-resolution Fourier transform ion cyclotron resonance mass spectrometry. J. Am. Soc. Mass Spectrom..

[23] Gomelsky M, Kaplan S (1997). Molecular genetic analysis suggesting interactions between AppA and PpsR in regulation of photosynthesis gene expression in Rhodobacter sphaeroides 2.4.1. J. Bacteriol..

[24] Winkler, A., Heintz, U., Lindner, R., Reinstein, J., Shoeman, R.L., Schlichting, I.: A ternary AppA-PpsR-DNA complex mediates light regulation of photosynthesis-related gene expression. Nat. Struct. Mol. Biol. **20**, 859–867 (2013) 10.1038/nsmb.2597 PMC3702404 23728293

[25] Senko MW, Beu SC, McLafferty FW (1995). Determination of monoisotopic masses and ion populations for large biomolecules from resolved isotopic distributions. J. Am. Soc. Mass Spectrom..

[26] Tibshirani R (1996). Regression shrinkage and selection via the lasso. J. Royal. Stat. Soc. (Series B).

[27] Efron B (2004). Least angle regression. Ann. Stat..

[28] Schwarz G (1978). Estimating the dimension of a model. Ann. Stat..

[29] Hamuro Y, Coales SJ, Molnar KS (2008). Specificity of immobilized porcine pepsin in H/D exchange compatible conditions. Rapid Commun. Mass Spectrom..

[30] Kirchner M, Xu B, Steen H, Steen JAJ (2011). libfbi: a C++ implementation for fast box intersection and application to sparse mass spectrometry data. Bioinformatics.

[31] Abzalimov RR, Kaltashov IA (2006). Extraction of local hydrogen exchange data from HDX CAD MS measurements by deconvolution of isotopic distributions of fragment ions. J. Am. Soc. Mass Spectrom..

[32] Morháč M (2006). Deconvolution methods and their applications in the analysis of gamma-ray spectra. Nucl. Instrum. Methods Phys. Res. A..

[33] Morháč M, Kliman J, Matoušek V (1997). Efficient one- and two-dimensional gold deconvolution and its application to γ-ray spectra decomposition. Nucl. Instrum. Methods Phys. Res. A..

[34] Morháč M, Matoušek V, Kliman J (2003). Efficient algorithm of multidimensional deconvolution and its application to nuclear data processing. Digital. Signal. Proc..

[35] Wu, J., van der Rest, G.: Improvement of Peptic Peptide Identification for Amide H/D Exchange using High Mass Accuracy Combined with a Statistical Approach. 61st ASMS Conference, Minneapolis, MN (2013)

[36] Weis DD, Wales TE, Engen JR (2006). Identification and characterization of EX1 kinetics in H/D exchange mass spectrometry by peak width analysis. J. Am. Soc. Mass Spectrom..

[37] The PyMOL Molecular Graphics System, version 1.2r3pre, Schrödinger, L.L.C. Available at: http://www.pymol.org/. Accessed 16 July (2012)

[38] Winkler A, Udvarhelyi A, Hartmann E (2014). Characterization of elements involved in allosteric light regulation of phosphodiesterase activity by comparison of different functional BlrP1 states. J. Mol. Biol..

