# Supplementary material for: Hexicon 2: Automated Processing of Hydrogen-Deuterium Exchange Mass Spectrometry Data with Improved Deuteration Distribution Estimation
Source: J Am Soc Mass Spectrom. 2014 Mar 28;25(6):1018–28. doi: 10.1007/s13361-014-0850-y (PMC4018513; doi:10.1007/s13361-014-0850-y)
Supplement: Supplementary file 1 — (PDF 935 kb) [file 13361_2014_850_MOESM1_ESM.pdf]

Supplemental Material

Hexicon 2: Automated Processing of Hydrogen-Deuterium Exchange Mass Spectrometry Data with Improved Deuteration Distribution Estimation.

Authors: Robert Lindner[1], Xinghua Lou[2], Jochen Reinstein[1], Robert L Shoeman[1], Fred A Hamprecht[2], Andreas Winkler[1,*]

Author Affiliations:
1: Department of Biomolecular Mechanisms, Max Planck Institute for Medical Research, Heidelberg, Germany

2: Heidelberg Collaboratory for Image Processing (HCI), University of Heidelberg, Heidelberg, Germany

Running Title: Hexicon 2: Automated Processing of HDX-MS Data

* Address correspondence to Dr. Andreas Winkler

Department for Biomolecular Mechanisms, Max Planck Institute for Medical Research, 69120 Heidelberg, Germany

Phone: +49 6221 486 508, Fax +49 6221 486 585,

E-mail: andreas.winkler@mpimf-heidelberg.mpg.de

Supplemental Figures

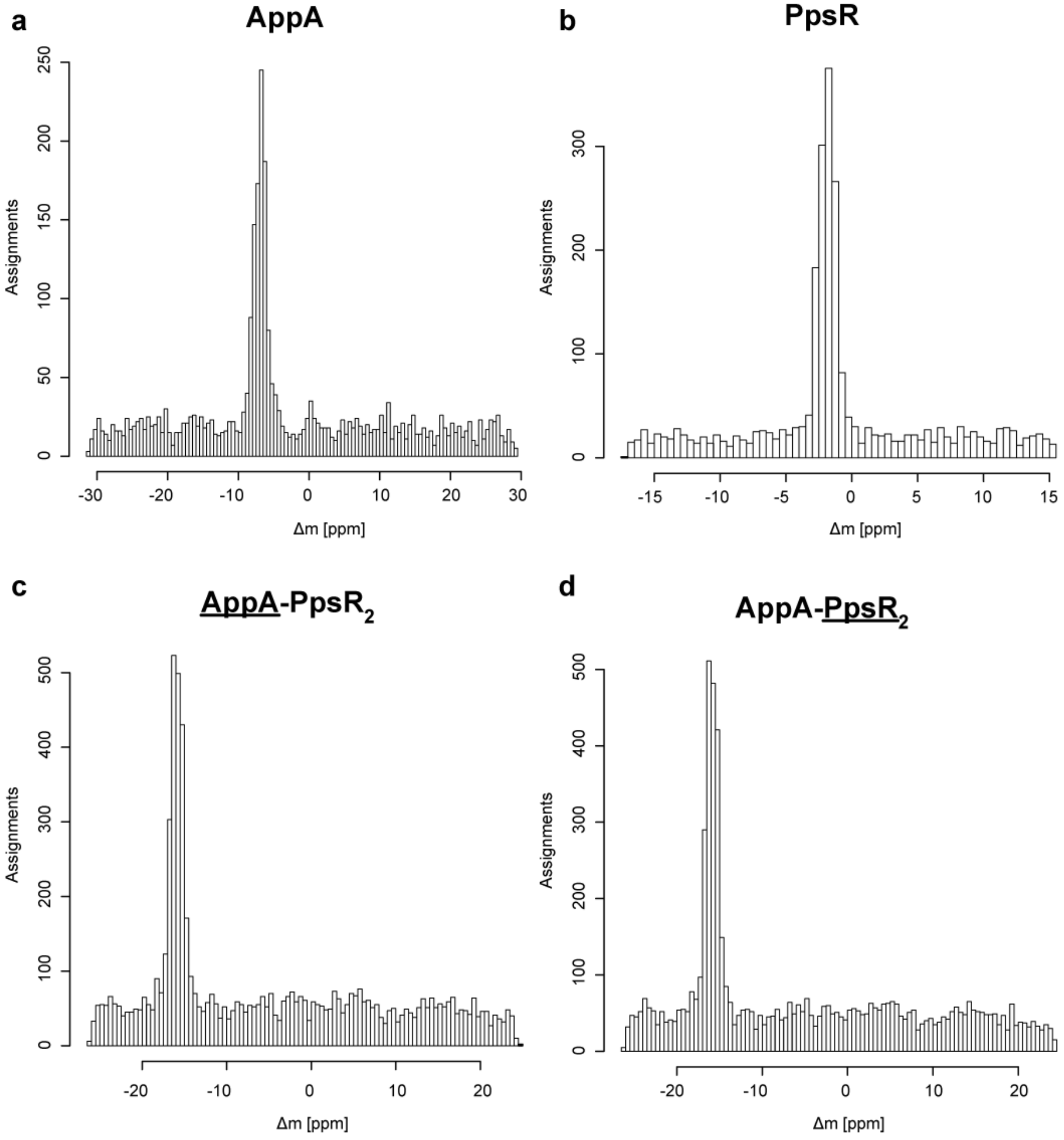

Supplemental Figure 1 Mass differences from sequence assignment. Relative differences between the observed mass and the theoretical mass from sequence assignment to peptides from AppA (a), PpsR (b) and AppA-PpsR$_2$ (c and d, for AppA and PpsR peptides, respectively) are shown. Correct sequence assignments to peptides from *in silico* digestion give rise to a peak in the histogram. The width and location of this peak indicate attainable mass precision and accuracy of calibration, respectively. The uniform assignment baseline is used to estimate the number of false positives within the peak area

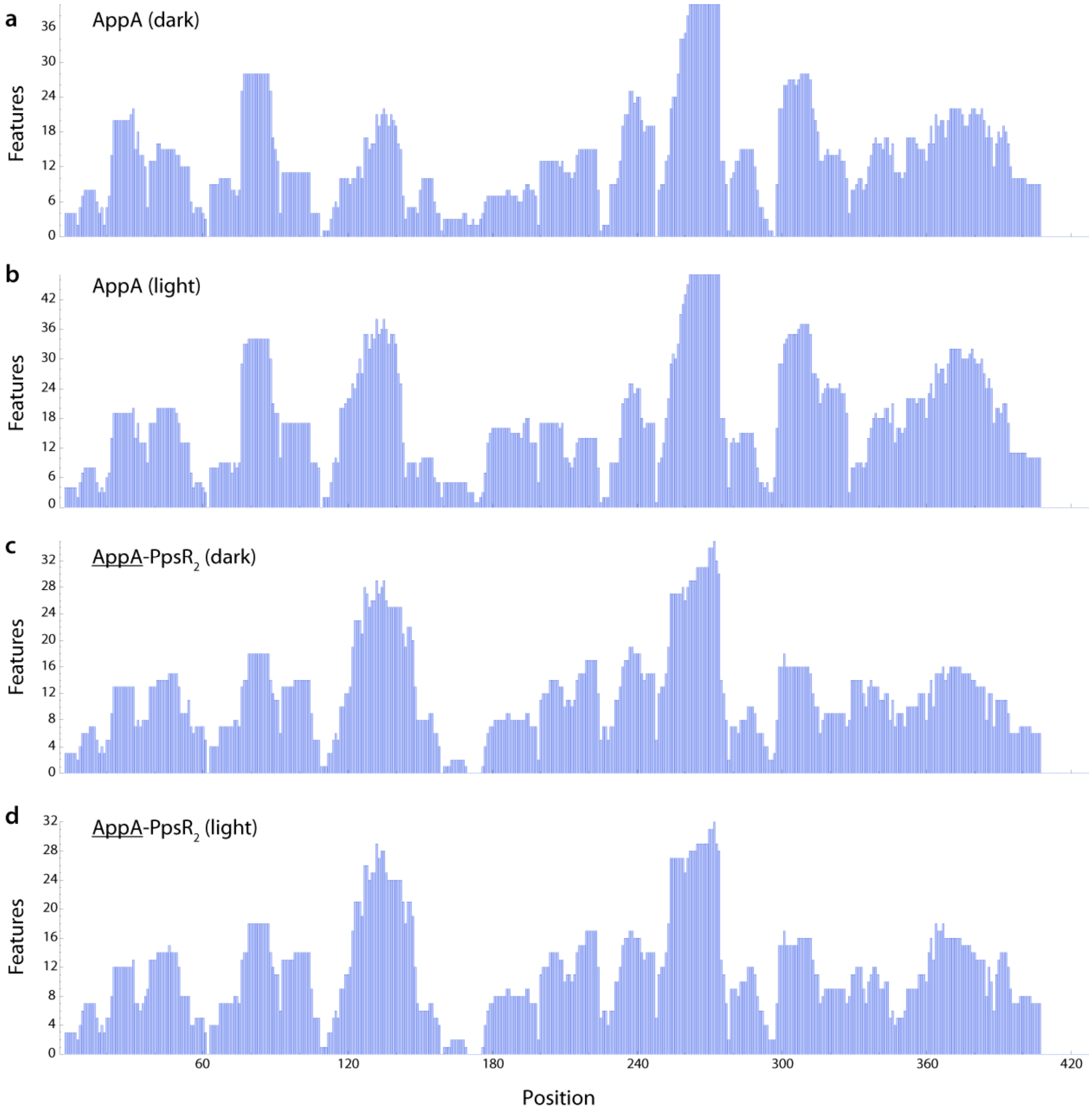

Supplemental Figure 2 Protein sequence coverage from Hexicon 2 analysis of AppA peptides. The histogram shows the number of features covering each amide in the protein backbone. (a) Free AppA in the dark state. (b) Free AppA in the light state. (c) AppA peptides in the dark state of the AppA-PpsR$_2$ complex. (d) AppA peptides in the light state of the AppA-PpsR$_2$ complex. Coverage is shown after automated and manual removal of poor quality results and taking into account that information from the N-terminal amide cannot be used due to rapid back-exchange under quench conditions

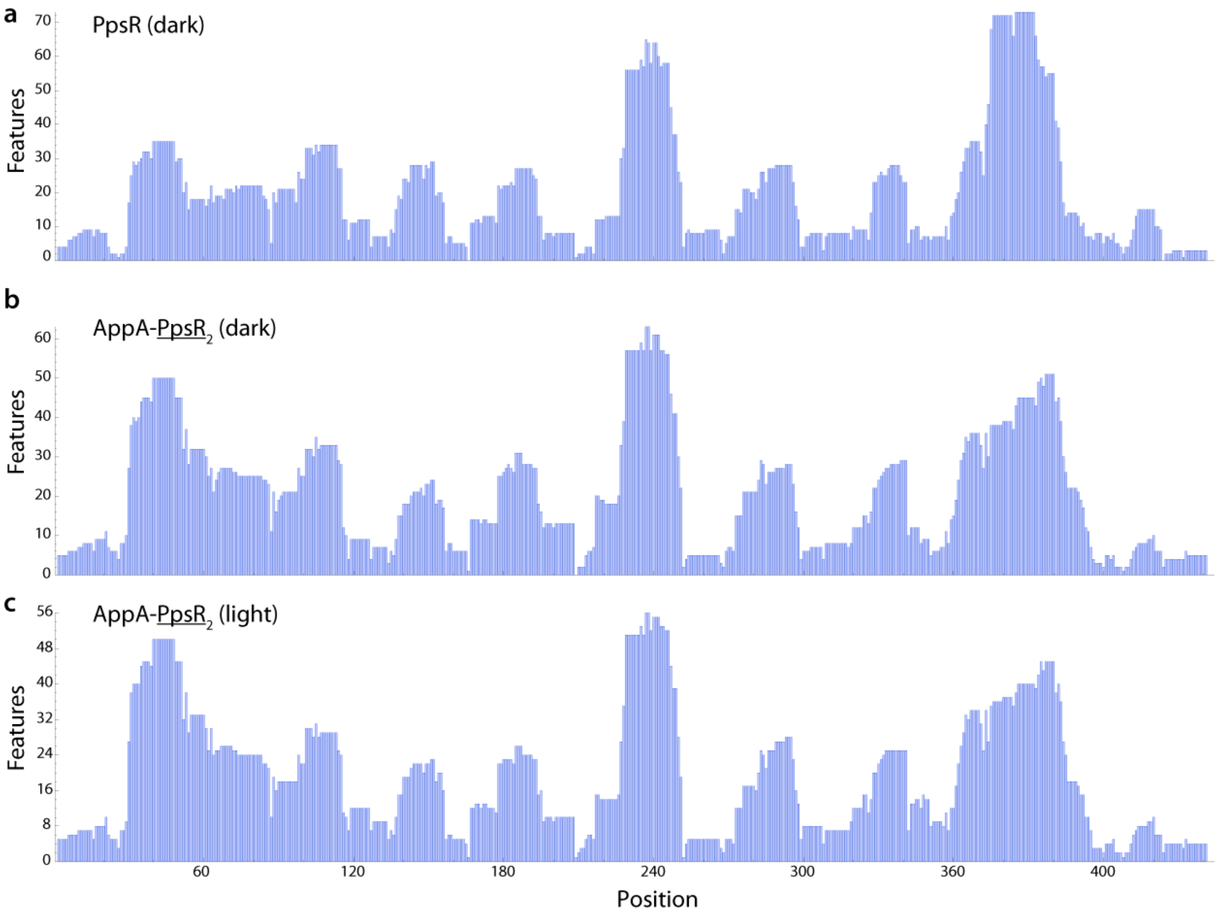

Supplemental Figure 3 Protein sequence coverage from Hexicon 2 analysis of PpsR peptides. The histogram shows the number of features covering each amide in the protein backbone. (a) Free PpsR. (b) PpsR peptides in the dark state of the AppA-PpsR$_2$ complex. (c) PpsR peptides in the light state of the AppA-PpsR$_2$ complex. Coverage is shown after automated and manual removal of poor quality results and taking into account that information from the N-terminal amide cannot be used due to rapid back-exchange under quench conditions

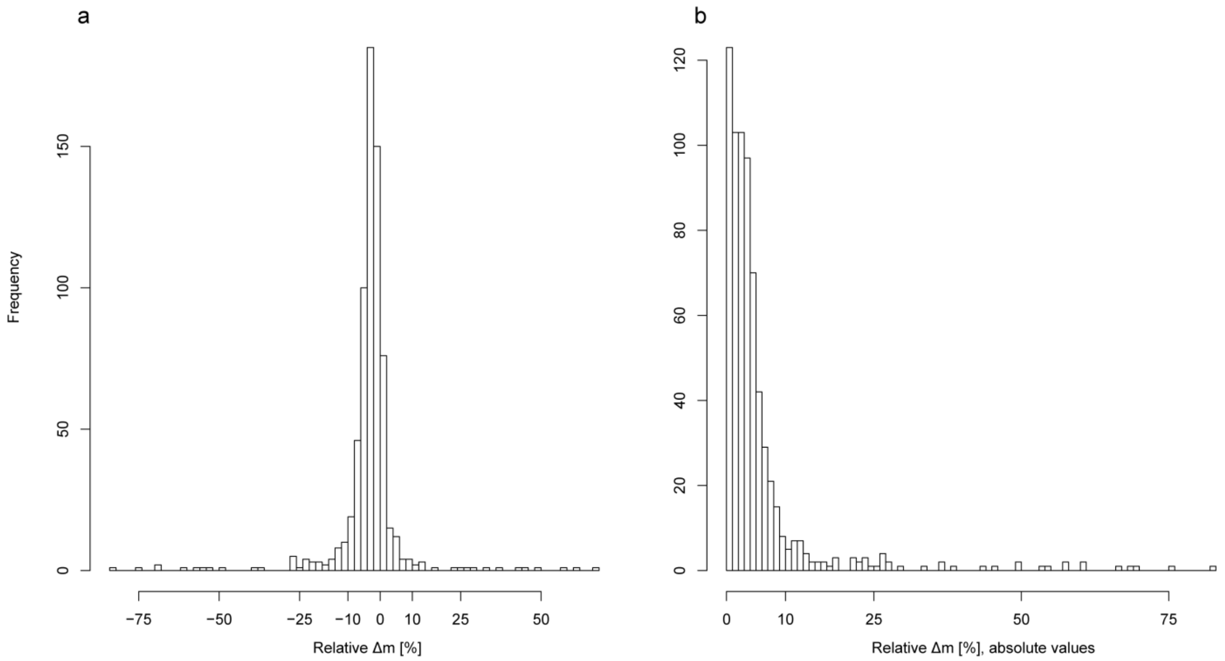

Supplemental Figure 4 Differences between deuteration centroids of Hexicon 2 and manual analysis. Deuteration centroids returned by Hexicon 2 and manual analysis using HX-Express [1] from 170 peptides and four deuteration time points each were evaluated. Panel a shows signed values, panel b shows a histogram of absolute values. The median absolute relative deuteration difference measures 3.1 % of the total deuteration, the 25th and 75th percentiles measure 1.5 % and 5.3 % of total deuteration, respectively

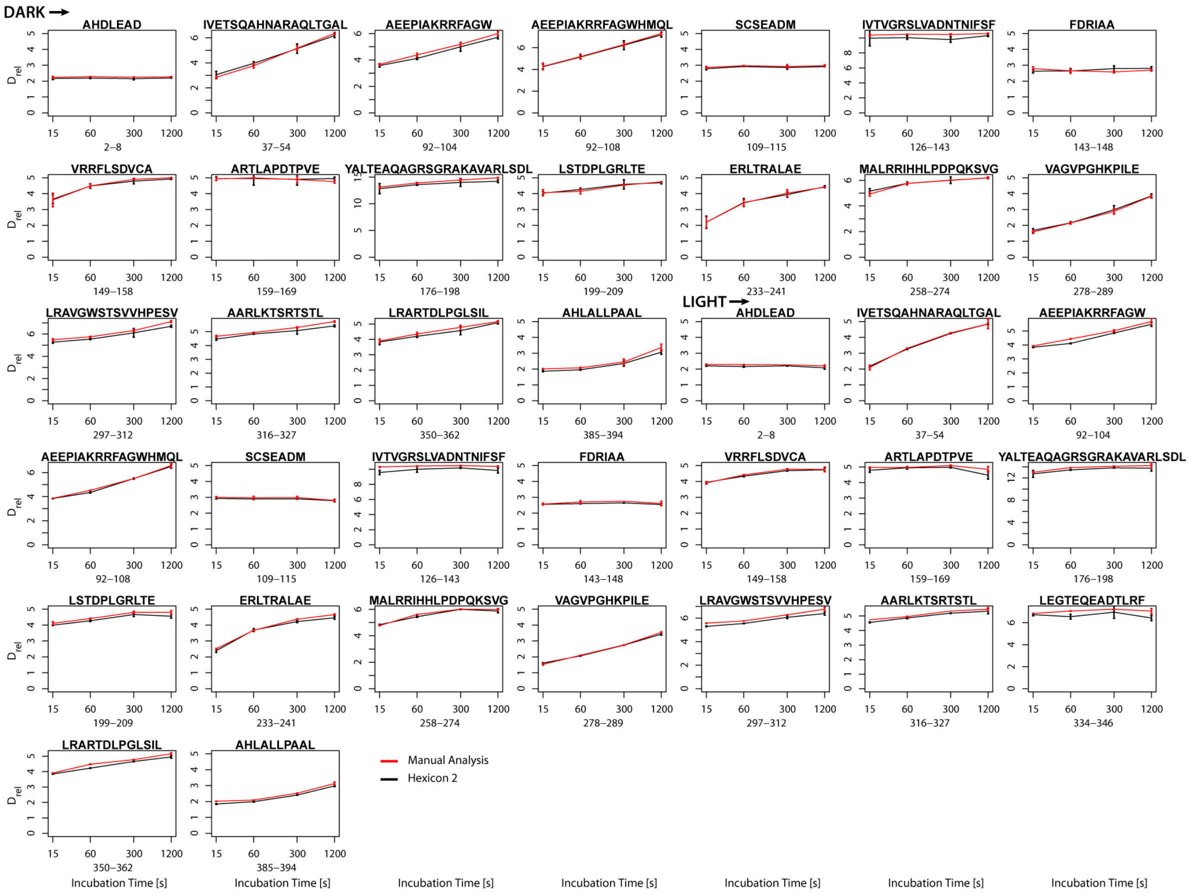

Supplemental Figure 5 Comparison of Hexicon 2 analysis of free AppA with manual evaluation. 37 peptides were commonly found in manual and automated analysis (18 from the dark state experiment and 19 from the light state experiment). Plots show relative deuterium incorporation over D_2O incubation time. Results from manual analysis are shown in red, Hexicon 2 results are shown in black

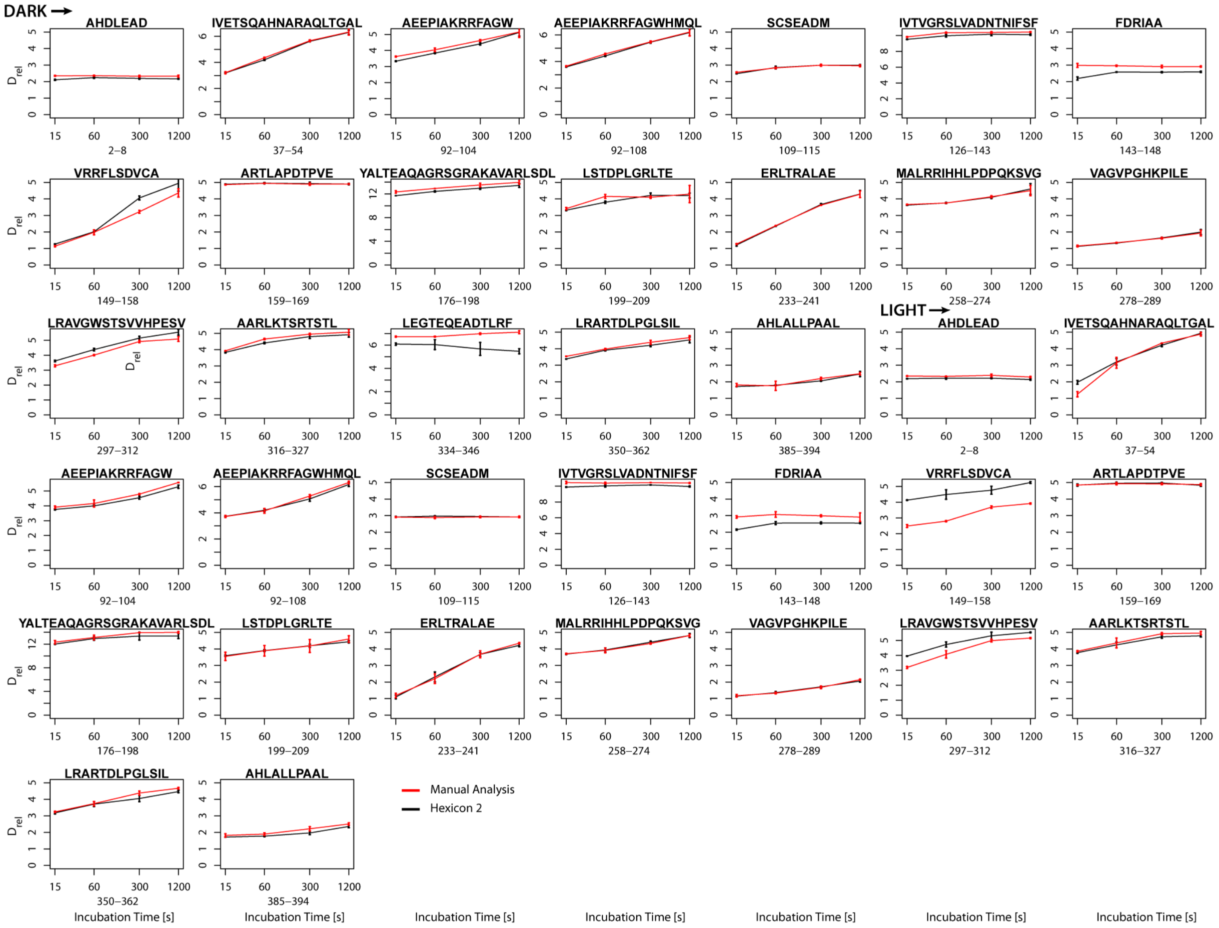

Supplemental Figure 6 Comparison of Hexicon 2 analysis of AppA peptides in the AppA-PpsR$_2$ complex with manual evaluation. 37 peptides were commonly found in manual and automated analysis (19 from the dark state experiment and 18 from the light state experiment). Plots show relative deuterium incorporation over D_2O incubation time. Results from manual analysis are shown in red, Hexicon 2 results are shown in black

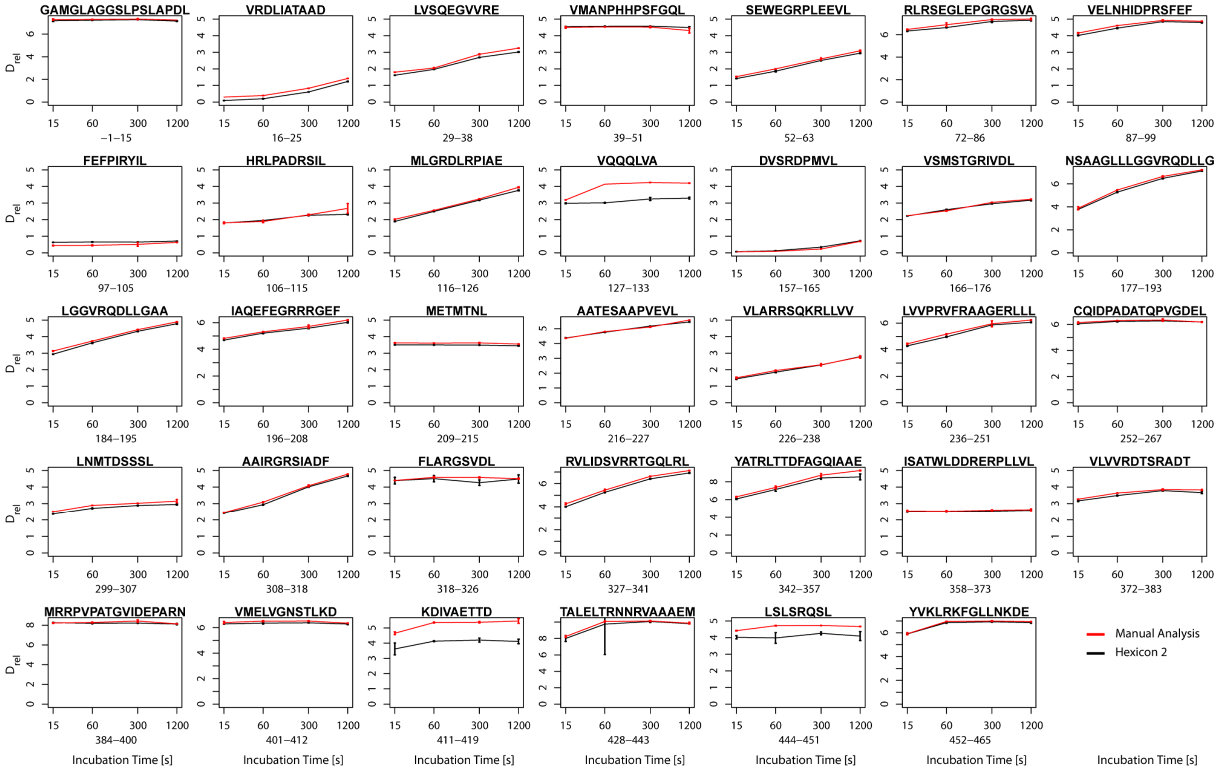

Supplemental Figure 7 Comparison of Hexicon 2 analysis of free PpsR with manual evaluation. 33 peptides were commonly found in manual and automated analysis. Plots show relative deuterium incorporation over D_2O incubation time. Results from manual analysis are shown in red, Hexicon 2 results are shown in black

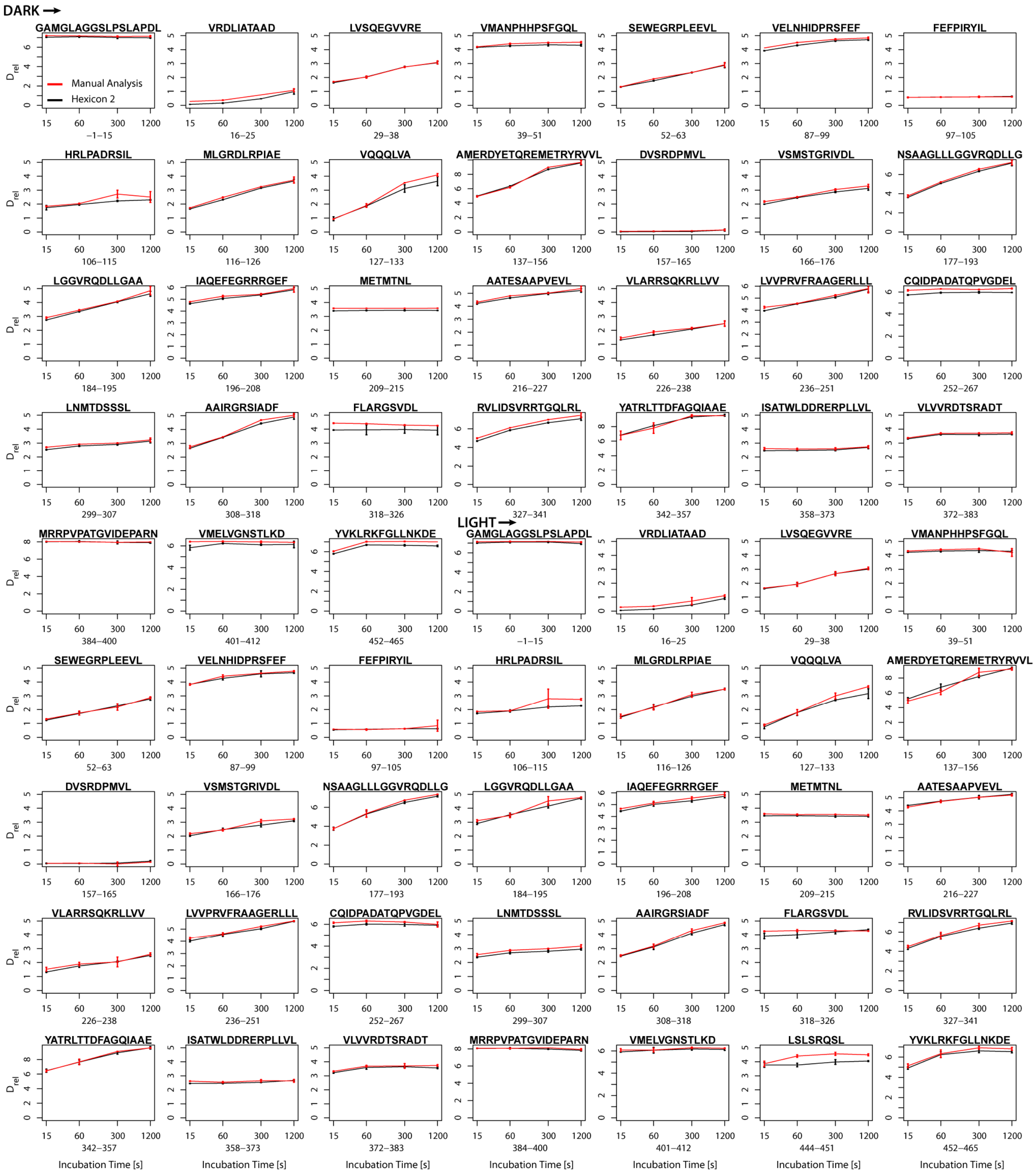

Supplemental Figure 8 Comparison of Hexicon 2 analysis of PpsR peptides in the AppA-$PpsR_2$ complex with manual evaluation. 63 peptides were commonly found in manual and automated analysis (31 from the dark state experiment and 32 from the light state experiment). Plots show relative deuterium incorporation over D_2O incubation time. Results from manual analysis are shown in red, Hexicon 2 results are shown in black

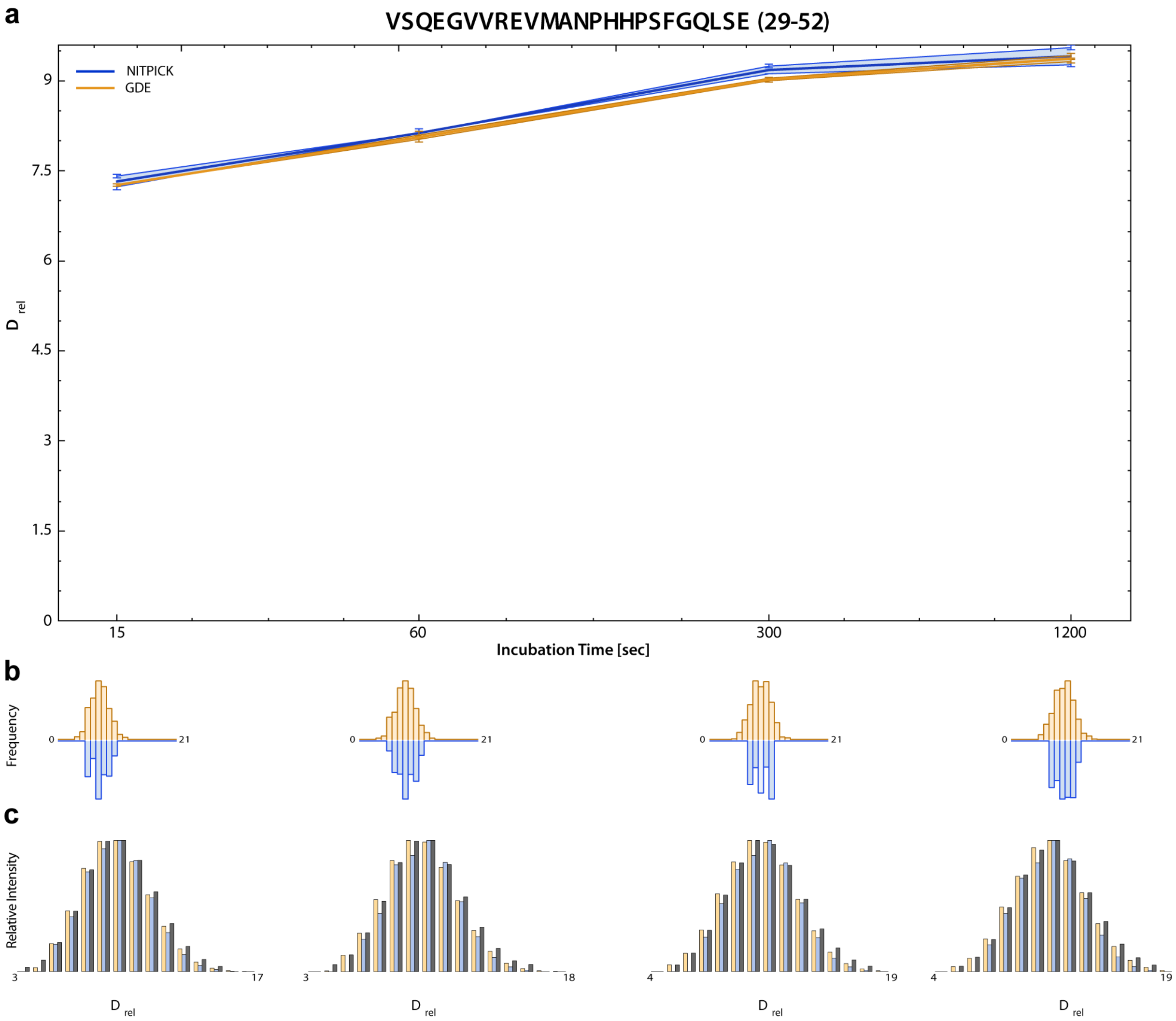

Supplemental Figure 9 Comparison of NITPICK with Gold iterative deconvolution (GDE) for deuteration distribution estimation. This example shows a case in which deuteration the centroids returned by NITPICK (blue) and GDE (orange) are nearly identical (a). Each bold line indicates a charge state of the shown peptide; the shaded area marks the span of deuteration values between charge states. NITPICK returns condensed distributions with only a few populated states around the centroid, whereas GDE returns a smooth distribution (b). Distribution estimates from both algorithms were used to reconstitute the corresponding observable isotope distribution in a mass spectrum (c). The actual observed mass spectrum is shown for comparison (panel c, gray bars)

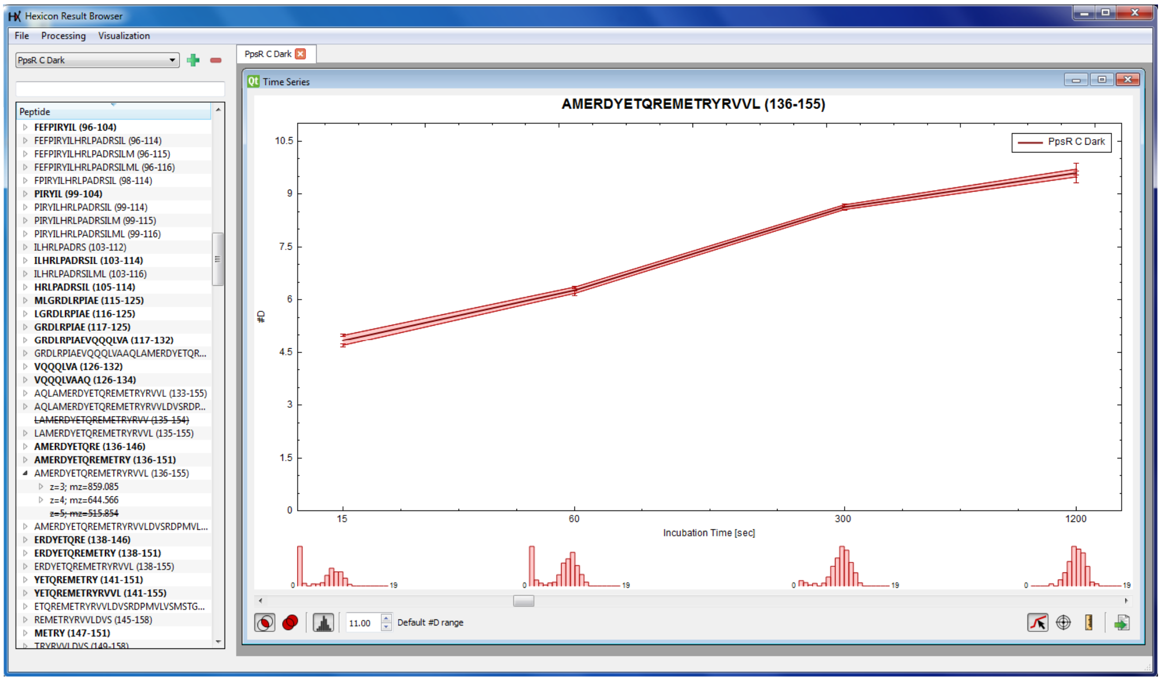

Supplemental Figure 10 Hexicon 2 result browser. Peptides are shown and can be manipulated in the tree view in the left part of the window. The right part shows different graphical representations of the data. In this case, the interactive time series view is shown in which the deuterium incorporation is plotted against incubation time. The bottom part of the plot shows the estimated distribution of deuteration states. Multiple peptides from one or more datasets can be loaded into the plot, deuteration values and differences can be measured and plots can be exported into PNG or PDF format

Online Resource 1: Retention Time Alignment in Hexicon 2

Hexicon 2 generates a global retention time mapping between the undeuterated reference and each map containing deuterated peptides (“target map“). The assumption that peptides elute in a largely fixed order allows a smooth global mapping rather than independent retention time prediction for each extracted feature.

In order to account for mass shifts through deuteration, each reference peptide is deuterated *in silico*, i.e., the monoisotopic mass is shifted by multiples of the H-D mass difference of approximately 1.0063 Da and used as search peaks. The actual isotope pattern is governed not only by H/D exchange but also by the natural occurrence of heavy isotopes. The mass difference between H/D isotopes and an average peptide’s isotope peaks is about 0.003 Da per isotope and may grow to measurable size. Since each peak in the observed isotope pattern contains a weighted average of isotope mass differences from the monoisotopic mass, the precise mass centroid of each isotope peak depends on the deuteration distribution which is not known *a priori* and the assumption that all centroids are to be found at multiples of the H/D mass difference from the monoisotopic mass consistently overestimates the measured mass centroids. Since there is only a limited number of natural heavy isotope peaks with appreciable intensity, the influence of the natural isotope pattern on the search peak centroid position is limited to the number of observable natural isotope peaks. Therefore, the all H/D mass difference is used as an upper bound and extended with a downwards window that depends on the number of observable natural isotopes in each peptide.

The noise baseline of each scan in the target map is determined using a running median and only peaks exceeding the baseline by a user-defined factor are kept. The fast box intersection library libfbi [2] is used to intersect the search peaks with the target map. Libfbi uses so-called box-generators to intersect boxes of arbitrary dimensions. Hexicon 2 defines retention time and and m/z as dimensions. Isotopes of deuterated models are surrounded by boxes spanning the entire retention time range and the expected m/z values of the isotope

pattern (see above). Peaks in the target map are surrounded by boxes matching the maximum expected calibration error provided by the user, thus, graphically, Hexicon 2 performs line intersection.

The intersections returned by libfbi are subsequently filtered to include only those spanning at least three adjacent scans representing a minimum number of consecutive isotopes (empirically determined as at least one fourth of the number of exchangeable amides, or six consecutive isotopes).

The filtered map is in most cases sufficient to create a coarse linear mapping using the median ratio of target retention time over reference retention time as slope and the median residual as intercept. Box intersections with regression residuals less than one standard deviation away from the linear mapping are kept as candidate mappings for the global modeling step.

The global model uses piecewise linear regression to generate a smooth mapping of reference to target retention times. This is done using a LOESS implementation adapted from the open source statistical software R (www.r-project.org), using a smoother span of one third and 20 iterations. Residuals of the LOESS model are locally smoothed using a Gaussian filter (vigra::recursiveGaussianFilterLine from the vigra library, https://hci.iwr.uni-heidelberg.de/vigra/) and stored for each time point as an estimate of the mapping precision.

In the last step, the expected retention time centroid of each feature is interpolated between the points of the LOESS fit. Since features elute within a time window, the centroid is extended by the reference elution time (i.e., the time difference between the first and the last scan in which the feature was detected) as well as the local smoothed LOESS residual (see above). A query of the alignment takes a retention time as parameter and returns all features predicted to contain the requested time in their elution windows.

A pseudocode representation of the alignment is given in listing 1.

Listing 1: Alignment algorithm used by Hexicon 2. A piecewise linear regression model is used to construct a global retention time mapping after intersection of the target map with mass positions predicted from *in silico* deuteration.

```
PeakBoxes ← list of SearchPeakBox
SpectralPeakBoxes ← list of SpectralPeakBox

for each species 𝒥 ∈ Reference {
        DeuteratedPeaks Φ_{D,𝒥} ← shiftIsotopePattern(𝒥)
        PeakBox PB(Φ_{D,𝒥}, mzSpan=isotopeError*numIsotopes, rtSpan=∞)
        PeakBoxes.add(PB)
}
for each scan s ∈ Target {
        Peaks p ← findSignificantPeaks(s)
        SpectralPeakBox SP(p, mzSpan=0, rtSpan=calibrationError)
        SpectralPeaks.add(SP)
}

Intersections ← libfbi.intersect(SpectralPeakBoxes, PeakBoxes)
intersectingLines ← list of fbi intersections
for each intersection 𝑥 ∈ Intersections {
        intersectingLines.add(𝑥,groupBy=PeakBox)
}

intersectingLines.keepOnlyConsecutiveScans

elutingIsotopePatterns ← list of intersectingLines
for each line ℓ ∈ intersectingLines {
        elutingIsotopePatterns.add(ℓ, groupBy=referenceSpecies)
}

elutingIsotopePatterns.keepOnlyConsecutiveIsotopes


preMap_slope ← median(elutingIsotopePatterns.targetRT / elutingIsotopePatterns.referenceRT)
preMap_intercept ← median(elutingIsotopePatterns.targetRT
                          – elutingIsotopePatterns.referenceRT * preMap_slope)
preMap_r ← getResiduals(elutingIsotopePatterns, preMap)
elutingIsotopePatterns.remove(preMap_r > standard_deviation(preMap_r))


globalMod ← LOESSModel
{mod, res} ← globalModel.fit(elutingIsotopePatterns)
toleranceWindow ← {elutingIsotopePatterns.referenceRT, res}
sort(toleranceWindow, by=rt)
GaussianSmooth(toleranceWindow)

PredictedTargets Ψ
for each species 𝒥 ∈ Reference {
        species ψ ← 𝒥
        ψ.rt ← mod(𝒥.rt)
        ψ.tolerance ← 𝒥.toleranceWindow + 𝒥.elutionTime / 2
        Ψ. add(ψ)
}

QueryAlignment(t)
        return { ψ ∈ Ψ |ψ. rt − ψ. tolerance < t < ψ. rt + ψ. tolerance }
```

References

1. Weis DD, Engen JR, Kass IJ (2006) Semi-automated data processing of hydrogen exchange mass spectra using HX-Express. J Am Soc Mass Spectrom 17:1700–1703. doi: 10.1016/j.jasms.2006.07.025

2. Kirchner M, Xu B, Steen H, Steen JAJ (2011) libfbi: a C++ implementation for fast box intersection and application to sparse mass spectrometry data. Bioinforma Oxf Engl 27:1166–1167. doi: 10.1093/bioinformatics/btr084
